# Flower-like ferro-polydopamine nanozymes with peroxidase-like activity for early caries prevention

**DOI:** 10.3389/fmicb.2026.1870312

**Published:** 2026-06-11

**Authors:** Pei Wang, Ziqiang Chen, Yifan Liu, Yuqing Mu, Jun Guo

**Affiliations:** 1 School of Stomatology, Jiangxi Medical College, Nanchang University, Nanchang, China; 2 Jiangxi Provincial Key Laboratory of Oral Diseases, Nanchang, China; 3 Jiangxi Provincial Clinical Research Center for Oral Diseases, Nanchang, China; 4 School of Medicine and Dentistry, Griffith University, Gold Coast, QL, Australia

**Keywords:** antimicrobial, dental caries, ferro-polydopamine, nanozyme, peroxidase-like activity

## Abstract

**Introduction:**

Dental caries, a highly prevalent oral disease, significantly compromises oral health. Dental biofilms represent a key etiological factor in caries pathogenesis and pose challenges for eradication due to their protective extracellular matrix.

**Methods:**

Herein, we rationally designed a ferro-polydopamine nanozyme (FPN) featuring a flower-like morphology and remarkable peroxidase-like (POD-like) activity.

**Results:**

Significantly, FPN efficiently catalyzed low-concentration H_2_O_2_ under mildly acidic conditions, exhibiting potent antibacterial activity against *Streptococcus mutans* (*S. mutans*) and robust anti-biofilm efficacy *in vitro*. Furthermore, enamel disks treated synergistically with FPN and low-dose H2O2 demonstrated significantly inhibited enamel demineralization compared to untreated controls under acidogenic challenge, indicating FPN’s remarkable preventive capacity against early caries.

**Discussion:**

Collectively, this study demonstrates that FPN, enabled by its superior POD-like activity and biocompatibility, holds substantial translational potential for significantly reducing clinical H_2_O_2_ concentrations while effectively preventing early dental caries.

## 1 Introduction

Dental caries, commonly known as tooth decay or cavities, represents the primary etiological factor behind toothache (Cheng et al., 2022). The pathological process involves a progressive destruction of dental hard tissues driven by acidogenic bacterial infection in the oral cavity (Zhang et al., 2022). Among these pathogens, *Streptococcus mutans* (*S. mutans*) adhering to the tooth surface is the primary etiological agent of dental caries, by inducing enamel demineralization and ultimately leading to cavitation (Thayumanavan et al., 2025). The irreversible cavity from this acid-induced demineralization triggers a spectrum of symptoms ranging from mild dentin hypersensitivity to severe pulpitis, periapical abscess, and even systemic infections (Kashyap et al., 2023). Consequently, the burden of dental caries remains a major global public health challenge, affecting more than 2.5 billion people worldwide (Benzian et al., 2025; Jevdjevic and Listl, 2025). The cornerstone of caries treatment involves the thorough removal of pathogenic biofilms, traditionally achieved through a combination of antibiotic therapy and mechanical debridement (e.g., scaling and ultrasonic cleaning). However, the widespread use of antibiotics has led to a crisis of drug resistance (Zhang et al., 2025). Meanwhile, mechanical methods are invasive, may cause irreversible damage to healthy tooth tissues. Hence, there is an urgent need to develop novel antibacterial strategies that are more efficient, targeted, and minimally invasive. This has become a central focus of current research efforts. To address the limitations of traditional therapeutic approaches, researchers have actively explored emerging antibacterial strategies in recent years, such as antimicrobial peptides, phage therapy (Hosseini Hooshiar et al., 2024; Zhu et al., 2025), and photodynamic therapy. However, these strategies still face bottlenecks including high cost, poor stability under oral conditions (acid production by cariogenic biofilms), or the need for complex equipment. The nanozymes have emerged as a research focus for the treatment of oral cariogenic bacterial infections, owing to their enzyme-mimetic catalytic activity, high stability, and tunability (Liang and Yan, 2019; Zandieh and Liu, 2024). Under acidic conditions, the peroxidase- and superoxide dismutase-like nanozymes catalyze the decomposition of H_2_O_2_ to generate highly reactive hydroxyl radicals (^⋅^OH) or superoxide anions (O_2_^–^), thereby reducing the clinical dosage of hydrogen peroxide and effectively disrupting microbial metabolism and biofilm structure (Gao et al., 2016). In addition, nanozymes containing metal ions (e.g., Fe^2+^/^3+^, Cu^2+^, Zn^2+^, Ag^+^) can release these ions to killing bacteria, which bind to bacterial DNA, proteins, or enzymes (Karim et al., 2018). Huang et al. developed a system where glucose oxidase linked to dextran-coated iron oxide nanozymes produces H_2_O_2_ from glucose. In acidic caries conditions, these iron oxide nanoparticles, exhibiting peroxidase-like activity, convert H_2_O_2_ into reactive oxygen species (Huang et al., 2025). Nevertheless, the application of existing nanozymes in the oral biomedical field still faces several challenges, such as the biosafety concerns by the reliance on heavy metals, the high cost of complex multi-step synthesis processes, and stringent preparation conditions and low production efficiency.

Iron-based nanozymes are a category of nanomaterials with iron as the core component, which effectively avoids the reliance on noble metals or rare-earth materials. According to differences in composition and structure, iron-based nanozymes can be classified into inorganic and organic types. Inorganic iron-based nanozymes (e.g., Fe_3_O_4_, γ-Fe_2_O_3_) have become a research hotspot owing to their high catalytic activity, magnetic responsiveness and low-cost advantages (Hwang et al., 2019). Organic iron-based nanozymes are complexes formed by the coordination of iron ions with organic ligands (e.g., porphyrins, polymers) enabling the catalytic activity and biocompatibility (Liu et al., 2024). The introduction of organic ligands (e.g., polydopamine, biopolymers) effectively reduces the free toxicity of iron ions, enhances the affinity between the material and biological interfaces, and decreases cytotoxicity and immunogenicity, making them more suitable for environments with stringent biosafety requirements (Jiang et al., 2026; Zheng et al., 2026). In addition, the synthesis of organic iron-based nanozymes is mild by the self-assembly and coordination precipitation without harsh conditions such as high temperature and high pressure, which better meets the demands of large-scale production (Liu F. et al., 2023; Ye et al., 2024). Meanwhile, there still hold a distinct cost advantage over noble metal-based nanozymes in terms of raw material expenses. Wu and colleagues describe a CWME-responsive nanozyme called F@Gala, created through the coordination of Fe^3+^ with the bioactive flavonoid galangin, which efficiently catalyzes the conversion of endogenous hydrogen peroxide into bactericidal hydroxyl radicals in the acidic microenvironment of infectious chronic wounds during early infection (Wu et al., 2026).

**SCHEME 1:**
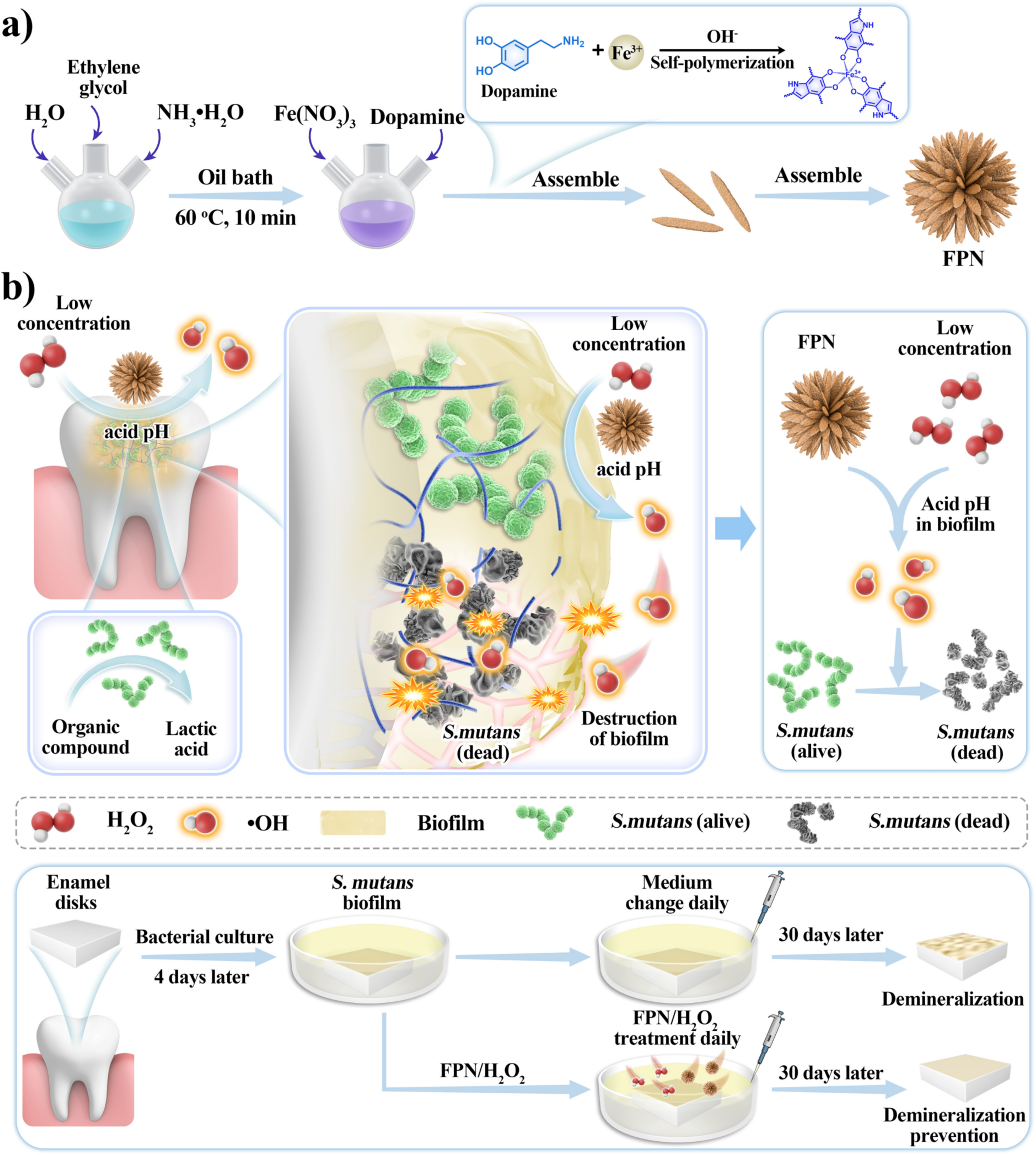
Schematic illustration of **(a)** the preparation process of the designed FPN by copolymerization of dopamine and Fe(NO_3_)_3_ and **(b)** the working mechanisms of FPN killing the *Streptococcus mutans* by generating hydroxyl radicals and preventing enamel demineralization.

Herein, to address the limitations of conventional nanozymes, we successfully fabricated flower-like ferro-polydopamine nanozymes (FPNs) through a facile and efficient one-step synthesis (Scheme 1). The synthesis process involves the initial coordination of dopamine with iron ions to form precursors, which are subsequently polymerized into nanoneedles in alkaline conditions under the regulation of a mixed ethylene glycol-water solvent. The as-formed nanoneedles further self-assemble into microscale flower-like FPN through intermolecular interactions including electrostatic forces, π–π stacking and hydrogen bonding of polydopamine. Such a hierarchical structure presents multiple superiorities: the ultrahigh specific surface area and multilevel porosity greatly increase the density of active sites and accelerate the rapid diffusion of substrate molecules to internal catalytic centers, thereby guaranteeing the efficient degradation of deep-seated biofilms. As an iron-based nanozyme, FPNs demonstrate exceptional catalytic activity in the acidic environment characteristic of cariogenic biofilms, efficiently decomposing H_2_O_2_ into highly reactive ^⋅^OH radicals. This enables precise eradication of Streptococcus mutans at low H_2_O_2_ concentrations, minimizing potential damage to healthy tissues. Furthermore, the organic-inorganic hybrid nature of FPNs confers both remarkable stability and excellent biocompatibility. To verify the designed functionality, we conducted systematic characterization using SEM, TEM, and XRD to analyze morphology and structural properties. Additionally, catalytic activity tests, antibacterial assays, stability analyses, and cytotoxicity evaluations were performed to confirm that FPNs exhibit both high catalytic efficiency and excellent biocompatibility in simulated oral environments. This structure-function synergistic design enables FPN to overcome the stability and permeability limitations of conventional nanozymes, offering an innovative solution for the eradication of recalcitrant cariogenic biofilms that combines high catalytic efficiency, deep penetration, and biocompatibility.

## 2 Materials and methods

### 2.1 Materials

All the chemical agents were analytical reagents (AR). Dopamine hydrochloride (DA), 3,3’,5,5’-tetramethylbenzidine (TMB), and brain-heart infusion broth (BHI) were obtained from Macklin Biochemical Technology Co., Ltd. (Shanghai, China). Hydrogen peroxide (H_2_O_2_) solution, ammonia water (NH_3_^⋅^H_2_O), ethylene glycol, acetic acid (HAc), sodium acetate (NaAc) and anhydrous ethanol were purchased from Xilong Science Co., Ltd. (Guangdong, China). Sucrose, sodium acetate, acetic acid, and 2.5% glutaraldehyde were purchased from Aladdin Biochemical Technology Co., Ltd. (Shanghai, China). Cell Counting Kit-8 reagent and dimethyl sulfoxide (DMSO) were obtained from Beyotime Biotechnology (Shanghai, China). Fetal bovine serum and trypsin digestion solution were purchased from Xinsaimei Biotechnology Co., Ltd. (Jiangsu, China).

### 2.2 Preparation of FPN

Briefly, FPN was synthesized by copolymerization of dopamine and ferric nitrate. Firstly, ethylene glycol and water were mixed in a ratio of 8:5 with stirring for 10 min. Then, 3 mL ammonia water was added to the ethylene glycol mixture solution. The mixture solution was stirred with speed of 650 rpm at 60 °C for 10 min. After that, the prepared 10 mL dopamine hydrochloride solution (50 mg mL^–1^) and 10 mL ferric nitrate solution (17.75 mg mL^–1^) were added into the ethylene glycol mixture solution and reacted for 11 h. FPN were harvested via centrifugation and water washing.

### 2.3 Physiochemical characterizations

SEM micrographs of FPN were obtained by scanning electron microscopy (SEM, Sigma 300, ZEISS, Germany). The functional groups of FPN were detected and analyzed by Fourier transform infrared spectroscopy (FT-IR, Thermo Scientific Nicolet iS20, United States). XPS (Thermo Scientific K-Alpha, US) was employed to further characterize the composition of the elements in the FPN.

### 2.4 FPN enzyme-like activities assay

#### 2.4.1 Measurement ^⋅^OH produced from FPN

To investigate the catalytic mechanism, ^⋅^OH produced in the catalyzing reaction was detected by electron paramagnetic resonance (EPR, EMXplus, Bruker). Briefly, the aqueous solution consisted of 50 mM 5,5-dimethyl-1-pyrroline N-oxide (DMPO) as the free radical trapping agent, 30 μL of FPN, 200 mM H_2_O_2_, and 1,910 μL buffer (pH 4.5). Then, ^⋅^OH was detected by the EPR under darkness after the addition of DMPO to the test sample for 0 min and 10 min of the reaction.

#### 2.4.2 POD-like activity assay

The steady-state kinetic behavior of FPN was determined to evaluate their catalytic performance. The kinetic parameters of FPN were obtained according to the change of absorbance of TMB at 652 nm in 10 min by an ultraviolet−visible (UV-vis) spectrophotometer (V-750, JASCO, Japan). The reaction was processed at room temperature by adding different concentrations of H_2_O_2_ in 1,780 μL NaAc-HAc buffer (0.1 M, pH 4.5) which contains FPN in a final concentration of 1 μg mL^–1^. Then, TMB was added to a final concentration of 0.25 mM. Similarly, different concentrations of TMB were added in 1,780 μL NaAc-HAc buffer (0.1 M, pH = 4.5) which contains FPN in a final concentration of 1 μg mL^–1^. Then, H_2_O_2_ was added to a final concentration of 0.5 mM. Finally, the kinetic parameters K_M_ and V_max_ were obtained according to the Michaelis-Menten (Equation 1):


v=V×max[S]/(K+M[S]) (1)

where [S] is the substrate concentration, K_M_ is the Michaelis-Menten constant and V_max_ is the maximal reaction velocity.

### 2.5 Antibacterial assay

#### 2.5.1 Antibacterial assay of H_2_O_2_ against *S. mutans*

To explore the inhibitory effect of H_2_O_2_ on *S. mutans*, 30% H_2_O_2_ (9.79 M) solution was diluted in 10^–1^ M, 10^–2^ M, 10^–3^ M, 10^–4^ M, and 10^–5^ M, respectively. The above five different concentrations of H_2_O_2_ solution (0.5 mL) were incubated with *S. mutans* suspension bacteria (0.5 mL) at 37°C for 5 min. Then, 10 μL bacteria solution were dropped into BHI agar plate. The surviving bacteria in the groups were counted by bacteria colony forming unit (CFU) counting method.

#### 2.5.2 Antimicrobial performance of FPN-catalyzed H_2_O_2_ against planktonic *S. mutans*

To explore the antimicrobial effect of FPN-catalyzed H_2_O_2_ on *S. mutans* suspension, the following experiments were performed. FPN catalyzed different concentrations of H_2_O_2_ (10^–2^ M, 10^–3^ M, 10^–4^ M, and 10^–5^ M) to kill the *S. mutans*. In addition, FPN catalyzed the same concentration of H_2_O_2_ to kill the *S. mutans* at different pH buffer including 4.5, 5.5, 6.5, 7. Moreover, FPN catalyzed 1 mM H_2_O_2_ to kill the *S. mutans* at pH = 4.5. Briefly, the *S. mutans* bacteria (0.3 mL) were incubated with FPN (0.3 mL, 0.5 mg mL^–1^) and H_2_O_2_ solution (0.3 mL) in a 37°C incubator for 5 min, Then, 10 μL bacteria solution were dropped into BHI agar plate. The surviving bacteria in each group were counted by CFU counting. Furthermore, the morphology of *S. mutans* in the FPN/H_2_O_2_ group, blank control group, H_2_O_2_ group, and FPN group were observed respectively by SEM to compare the change in the morphology of bacteria more visually.

#### 2.5.3 Anti-biofilm performance of FPN-catalyzed H_2_O_2_ against *S. mutans*

To construct bacterial biofilms of *S. mutans* on hydroxyapatite (HA) disks, the following approaches were employed. HA slices were soaked in the sterile saliva for 2 h at 37°C. Then, the HA disks covered with saliva film were placed in a 48-well plate at 37 °C and anaerobic conditions, and 1 mL of BHI culture medium containing 1% (w/v) sucrose and 1 × 10^5^ CFU/mL of *S. mutans* was added. After that, the fresh BHI culture medium was replaced every 24 h for a period of 4 days to form biofilm.

To explore the anti-biofilm concentration of H_2_O_2_ for *S. mutans* biofilm, 10^–1^ M, 10^–2^ M, 10^–3^ M, and 10^–4^ M H_2_O_2_ was catalyzed respectively by FPN. After removing the BHI medium, 0.5 mL FPN (0.5 mg mL^–1^) at pH 4.5 buffer and 0.5 mL H_2_O_2_ solution were added into 48-well plate with HA disks. After incubation for 10 min at 37°C in an incubator, the HA disks were washed 3 times with sterile water. Then, each group of HA disks was placed in a centrifuge tube with 1 mL of BHI medium. The ultrasonic oscillation was performed for 5 min before conducting CFU plate counting. The antibiofilm activity in each group was counted by the CFU counting method. After selecting the appropriate H_2_O_2_ concentration, the remaining biomass of the bacterial biofilm was further explored in the blank control group, the H_2_O_2_ group, the FPN group, and the FPN/H_2_O_2_ group by CFU counting. In addition, the Live and Dead Viability/Cytotoxicity Assay Kit was used to stain bacteria. Then the images of biofilms were observed by a confocal laser scanning microscope (CLSM, A1 HD25, Nikon, Japan).

### 2.6 The biocompatibility

#### 2.6.1 The cellular biocompatibility

Briefly, human gingival fibroblasts (HGFs) were seeded into 96-well plates with 6,000/well and incubated for 24 h. The collection and use of HGFs were approved by the Ethics Committee of the Affiliated Stomatological Hospital of Nanchang University (Approval No. 2024-50). Later, a fresh medium containing various concentrations of FPN was replaced and incubated for 24 h. Finally, 10 μL of CCK-8 kit was added. After 2 h, the absorbance was measured at 450 nm by a microplate reader (Infinite 200 Pro, Tecan, Austria). Cell viability rate was calculated according to the following formula (Equation 2):


Cellviabilityrate(%)=(A-treatmentA)blank/(Acontrol (2)

-A)blank×100%

where A_treatment_ is the absorbance of the treatment group and A_blank_ is the absorbance of the blank group, and A_control_ is the negative control group.

#### 2.6.2 The hemolysis test

To test the hemolysis rate of FPN, the erythrocytes from Wistar rats fresh blood were collected, which were performed strictly under the Animal Management Rules of the Ministry of Health of the People’s Republic of China and the Jiangxi Medical Device Testing Center Animal Care and Use Committee (PZ202302) and executed rigorously. Briefly, diluted erythrocyte suspension (0.2 mL) was mixed with saline (0.8 mL) as a negative control and water (0.8 mL) as a positive control. Then, 0.05, 0.1, 0.3, 0.5, 0.7, and 1.0 mg mL^–1^ FPN suspensions (0.8 mL) were prepared as experimental groups. All mixtures were kept at room temperature for 3 h. The treated samples were centrifuged and the absorbance of the supernatant at 541 nm was determined by UV-Vis spectrophotometry. Finally, the hemolysis rate was calculated according to the following formula (Equation 3):


Hemolysisrate(%)=(A-sampleA)negative/(Apositive (3)

-A)negative×100%

where A_sample_ is the absorbance of the FPN group and A_positive_ is the absorbance of the positive control group, and A_control_ is the negative control group.

### 2.7 Effect of FPN/H_2_O_2_ on tooth surface structure

Forty premolar teeth that had been extracted due to orthodontic treatment were collected, and the caries-free, morphologically normal teeth without enamel defects were selected and cleaned ultrasonically. The collection and use of human enamel specimens for this *in vitro* study was reviewed and approved by the Ethics Committee of the Affiliated Stomatological Hospital of Nanchang University (Approval No. 2024-50). Enamel-dentin disks of 4 mm × 4 mm size were cut at the center of the buccal surface of the premolar crowns using a hard tissue slicer (Exakt400cs, Germany). The enamel disks were randomly divided into 4 groups, and the Vickers hardness (HV) of enamel disks was measured by a micro-Vickers hardness tester (VH1202, Buehler, US) with a Vickers indenter load of 0.49 N and a pressurization time of 10 s. The hardness was measured at 4 points on the enamel disks, with each point separated by more than 1 mm.

After measuring the initial hardness of each enamel disk, *S. mutans* biofilm was grown on the surface of the enamel disks by the same above method. The treatment was started after the construction of bacterial biofilm in each group, and the medium was replaced every 24 h for 30 days. Each group was treated for 10 min before replacing the medium every 24 h. Control group was incubated with 1 mL of sterile water. FPN group was treated with 1 mL FPN (1 mg) solution. H_2_O_2_ group was treated with 1 mL of 2 × 10^–2^ M H_2_O_2_ solution. FPN/H_2_O_2_ group was treated with 1 mL of 2 × 10^–2^ M H_2_O_2_ solution and 1 mg of FPN. After 30 days of treatment, the enamel disks of each group were taken out and observed the surface morphology by SEM. Moreover, the Vickers hardness of the enamel disks was measured respectively.

### 2.8 Statistical analysis

All data were expressed as the mean ± standard deviation (*n* ≥ 3). The statistical significance of the data was calculated via a one-way analysis of variance (ANOVA) followed by Tukey’s test. A *p*-value of < 0.05 was considered statistically significant (*p < 0.05, **p < 0.01, ***p < 0.001).

## 3 Results and discussion

### 3.1 The physicochemical characterizations of FPN

**FIGURE 1:**
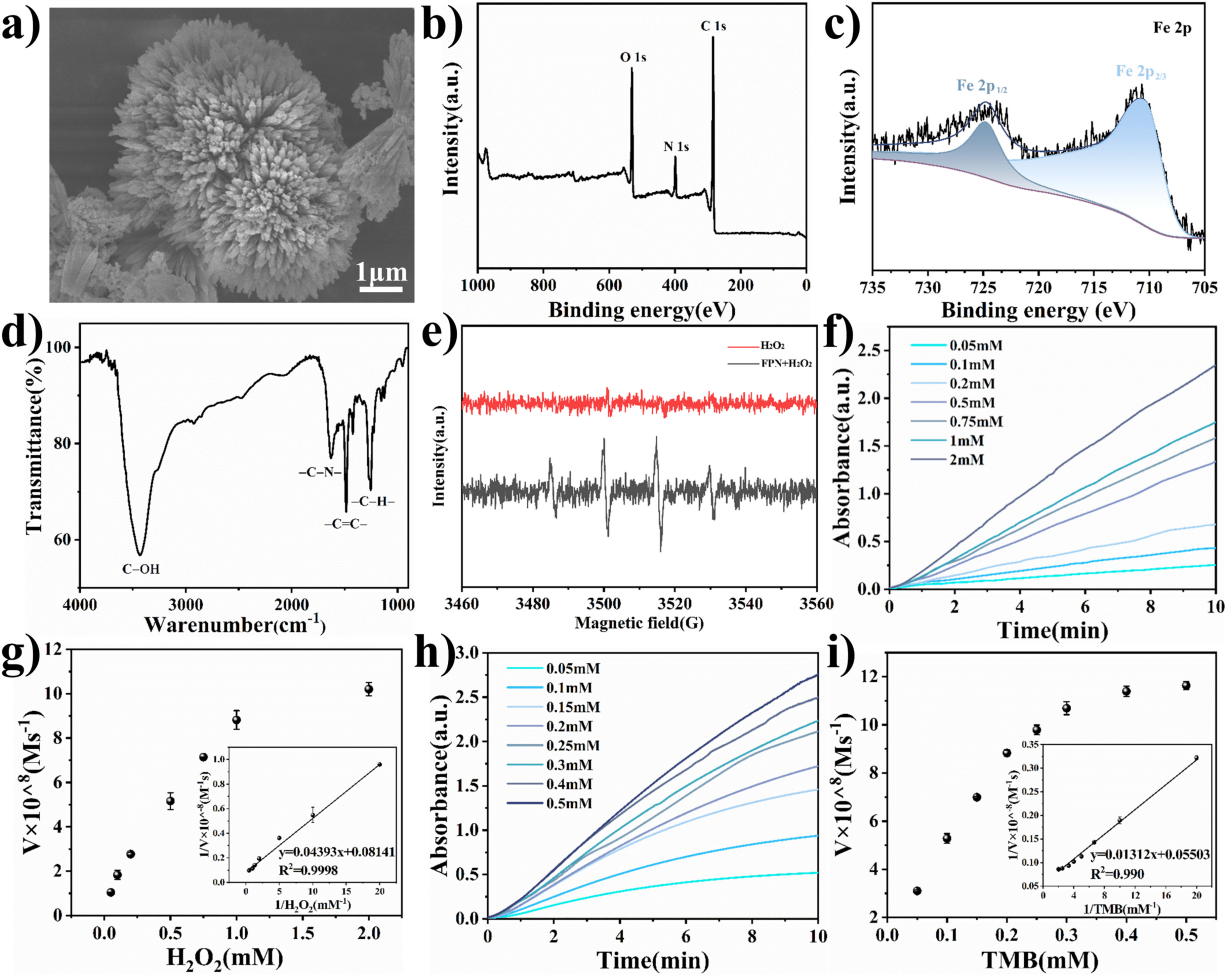
The physiochemical characterization and like-enzyme activities of the designed FPN. **(a)** SEM image showing the morphology of FPN. **(b)** X-ray photoelectron spectroscopy of FPN showing Fe, C, N, and O elements. **(c)** XPS spectra of Fe element at FPN. **(d)** FT-IR spectra of FPN presenting the covalent bonds. **(e)** The EPR characterization of FPN indicating the catalysis to produce ^⋅^OH from H_2_O_2_. **(f)** The absorbance of the oxidate TMB on the POD-like activity of FPN with various concentrations of H_2_O_2_. **(g)** Steady-state kinetic assay of FPN with different concentration of H_2_O_2_ varying from 0.05 to 2 mM. **(h)** The kinetic research of the POD-like activity of FPN under various concentrations of TMB. **(i)** Steady-state kinetic assay of FPN with different concentration of TMB varying from 0.05 to 0.5 mM.

To achieve the excellent biocompatibility Fe-based nanozyme applying for oral field, the natural molecule dopamine was employed as the ligand for organic iron-based nanozymes. Despite its good excellent biocompatibility, dopamine readily coordinates with metal ions including iron ions owing to catechol, which effectively mitigate the aggregation of iron-based nanozymes (Wang Y. et al., 2026). Furthermore, the self-polymerization of dopamine in an alkaline environment synthesized dopamine-iron nanoneedles, which further self-assemble to a flower-like microsphere with high specific surface area and hierarchical porosity. To observe the morphology and characterizations, SEM and other measures were examined for FPN. As shown in Figure 1a and Supplementary Figure S1, FPN obviously exhibited a flower-like and spherical morphology with approximately 4 μm diameter comprising numerous nanorods. The length of FPN nanorods ranged between 200 and 500 nm, and the width was about 50 nm. The FPN’s flower-like structure was derived from a metal-driven self-assembly strategy by tuning the volume ratio of ethylene glycol to water as our previous reports, comparing with general sphere morphology of ferric-polydopamine composites (Liu F. et al., 2023; Ye et al., 2024; Gao et al., 2025). In addition, the precoordination copolymerization approach enables a higher iron ions loading capacity compared with the postcoordination method, thereby delivering superior POD activity (Zhang et al., 2024; Gao et al., 2025).

To further investigate the elemental composition and the chemical bonds in FPN, XPS and FTIR were examined to verify the successful synthesis of the material. The XPS survey spectrum of FPN (Figure 1b) reveals distinct photoemission peaks corresponding to carbon (C 1s, ∼285 eV), nitrogen (N 1s, ∼400 eV), and oxygen (O 1s, ∼532 eV), indicating that PDA was effectively polymerized and participated in the synthesis of FPN. In contrast, an extremely weak signal of Fe peak was detected in the survey spectrum at the range of 705–735 eV. The plausible explanations of weak Fe signal are that the ferric species are encapsulated by the surface organic layer, or highly dispersed as small coordination complexes (Yuan et al., 2024). Meanwhile, the high-resolution XPS spectrum of Fe2p (Figure 1c) shows the Fe2p_3/2_ and Fe2p_1/2_ peaks at approximately 711.5 and 724 eV, accompanied by a weak satellite peak at around 718.5 eV, which are characteristic and shake-up satellite of Fe^3+^. The features confirm that Ferrum in FPN predominantly exists in the + 3-oxidation state. Regarding the weak satellite intensity compared to Fe^3+^ oxides, it is likely attributed to the coordination environment of Fe^3+^ with the catechol groups of PDA (Pang et al., 2026). As shown in Figure 1d, the FTIR spectrum of FPN exhibits characteristic absorption bands of PDA. A broad band centered at around 3,400 cm^–1^ is attributed to the O–H and N–H stretching vibrations of the catechol and amine groups, and the peaks at approximately 1,620 and 1,510 cm^–1^ correspond to the C = C stretching of the aromatic ring and the N–H bending vibration. The band at around 1,280 cm^–1^ is assigned to the C–N stretching vibration, while the peak at 1,100 cm^–1^ arises from the C–O stretching. In addition, the catechol O–H stretching band slightly shifts to lower wavenumbers, indicating the formation of Fe^3+^-catechol complexes, further confirming the successful incorporation of iron into the PDA network.

### 3.2 FPN enzyme-like activity

To verify whether our designed FPN exhibits enzyme-like activity through a Fenton-like reaction, EPR spectroscopy was employed to directly detect ^⋅^OH generation by using DMPO as a spin-trapping agent. As illustrated in Figure 1e, the H_2_O_2_ group displayed featureless baseline noise. After catalysis reaction, a characteristic signal of the DMPO-^⋅^OH adduct was observed at approximately 3,485, 3,500, 3,515, and 3,530 Gauss (G), demonstrating that FPN catalyzed H_2_O_2_ to generate ^⋅^OH by Fenton-like reaction as most iron-based nanozymes. Specifically, Fe^3+^ in FPN is partially reduced to Fe^2+^ by the catechol groups of polydopamine, and the regenerated Fe^2+^ reacts with H_2_O_2_ to produce ^⋅^OH via the classic Fenton reaction. The continuous cycling between Fe^3+^ and Fe^2+^ ensures sustained catalytic activity. In addition, it was noted that biocompatible PDA with reducible catechol did not limit ^⋅^OH production as other reports (Zhang et al., 2026). The plausible reasons are that the protonation of PDA’s catechol under acidic catalytic conditions markedly suppresses its reducibility, and the coordination between catechol with iron ions further diminishes its ^⋅^OH-scavenging ability. In addition, PDA could facilitate the reduction of Fe^3+^ to Fe^2+^, which maintains an efficient redox cycle for the benefit of the Fenton-like reaction.

To further explore the POD-like activity of FPN, the reaction kinetics were identified through the TMB chromogenic assay by measuring the absorbance at 652 nm, which serves as a standard substrate for horseradish peroxidase (HRP) or other peroxidases (Srinivasan, 2022). Kinetic assays were performed by monitoring the absorbance of oxidized TMB with various concentrations of H_2_O_2_ (0.05–2 mM) and TMB (0.05–0.5 mM) within 10 min, which Michaelis-Menten’s kinetic parameter (K_M_) and the maximum initial velocity (V_max_) were calculated by Lineweaver-Burk plots (Figures 1f–i). The values of K_M_ for TMB and H_2_O_2_ on FPN were 0.238 mM and 0.540 mM, and the V_max_ values were 1.82 × 10^–7^ M s^–1^ and 1.23 × 10^–7^ M s^–1^, respectively (Figures 1g,i). In general, a lower value of K_M_ that reflects the affinity between the enzyme and the substrate indicates a higher affinity of the enzyme for the substrate. The K_M_ value of the conventional peroxo-horseradish enzyme for H_2_O_2_ is approximately 2.528 mM (Lu et al., 2023), and the K_M_ value of Fe_3_O_4_ for H_2_O_2_ is about 154 mM (Gao et al., 2007), which indicated that FPN presented a stronger affinity for H_2_O_2_.

### 3.3 Antibacterial performance of FPN against planktonic *S. mutans*

**FIGURE 2:**
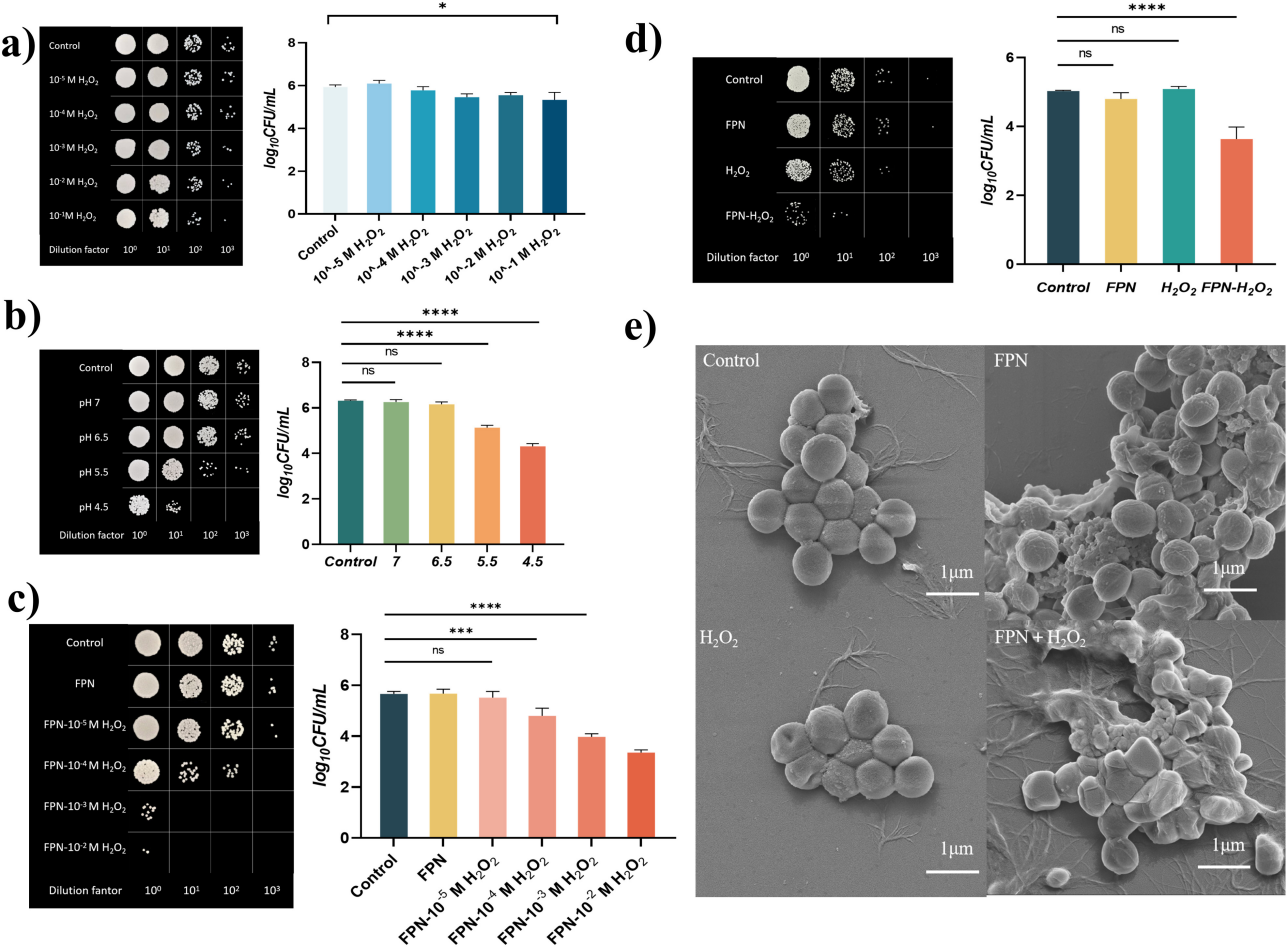
The antibacterial effect of FPN against planktonic *S. mutans.* **(a)** The antibacterial effects of different concentrations of H_2_O_2_ on planktonic *S. mutans*. **(b)** Effects of different pH on the bactericidal effect of 1 mM H_2_O_2_ catalyzed by FPN on planktonic *S. mutans*. **(c)** The antibacterial effect of 1 mM H_2_O_2_ catalyzed by FPN at pH = 4.5. **(d)** The effect of different concentrations of H_2_O_2_ catalyzed by FPN on *S. mutans* suspension. The left images of **(a–d)** are the CFU images and the right images are the CFU count. **(e)** SEM images of *S. mutans* after various treatments by H_2_O_2_ and H_2_O_2_/FPN. *P < 0.05, ***P < 0.001, ****P < 0.0001.

The prolonged clinical application of high-concentration H_2_O_2_ (∼1 M) is associated with adverse effects on healthy oral tissues, such as mucosal irritation and dysbiosis (Satti et al., 2023). To improve the oxidative efficiency and reduce the reliance on high concentrations and dosages of H_2_O_2_, we leveraged the efficient POD-like activity of FPN to catalyze low concentrations of H_2_O_2_ for ^⋅^OH generation, and thereby effectively killing cariogenic bacteria. To evaluate the bactericidal effect of H_2_O_2_ on *S. mutans* under varying concentration, the bacteria were exposed to H_2_O_2_ concentrations ranging from 0.3 × 10^–1^ to 0.3 × 10^–5^ M (Figure 2a) for 5 min. As illustrated in Figure 2a, the CFU images and corresponding quantitative analysis demonstrated that H_2_O_2_ alone at low concentrations had no discernible killing effect on *S. mutans*. It is partly because the concentration was substantially lower than clinical standards, combined with a low rate of ROS production.

Further, to measure the bactericidal effect on *S. mutans* by FPN catalyzing H_2_O_2_ under varying pH conditions and concentration, the bacteria were exposed to H_2_O_2_ concentrations at pH levels of 7.0, 6.5, 5.5, and 4.5 (Figure 2b) and ranging from 0.3 × 10^–1^ to 0.3 × 10^–5^ M (Figure 2c) for 5 min. As displayed at Figure 2b, bacterial CFUs at pH 4.5 decreased the number of surviving *S. mutans* by 2 orders of magnitude in suspension, in comparison to pH 7, 6.5, and 5.5, demonstrating the highest efficiency of FPN in catalyzing H_2_O_2_ at pH 4.5 approximately at which *S. mutans* produces acid (Chen et al., 2026). For catalyzing various concentration of H_2_O_2_ (Figure 2c), no statistically significant difference in bacterial viability was observed between the FPN alone group or the FPN/10^–5^M H_2_O_2_ group and the control group (*p* > 0.05). Specifically, starting from the FPN/10^–3^M H_2_O_2_ group, the bacterial count decreased by two orders of magnitude. In addition, the antibacterial effect of FPN-catalyzed H_2_O_2_ against *S. mutans* exhibited a clear concentration dependence, attributing to more ^⋅^OH generation at the same short time.

Based on the above evaluation, 10^–3^M (1mM) H_2_O_2_ which is approximately 1,000-fold lower than that of clinically used 3% H_2_O_2_, and pH4.5 corresponds to the acid-producing environment of *S. mutans*, were selected for subsequent experiments. For further investigating the antibacterial efficacy and observing the morphological changes of *S. mutans*, CFU assays and SEM observations were performed under these conditions (Figures 2d,e). It was found that under slightly acidic conditions (pH4.5), FPN catalyzing 1 mM H_2_O_2_ could reduce the number of surviving *S. mutans* in suspension by 2 log, exhibited a notable planktonic antibacterial effect (Figure 2d). In contrast, neither the H_2_O_2_ group alone nor the FPN group alone showed significant antibacterial activity, and their viable bacterial counts were not statistically different from that of the blank control group. Furthermore, the morphological changes of *S. mutans* in the FPN/H_2_O_2_, FPN, the H_2_O_2_ group, and the blank control groups were observed by SEM. Compared with the blank control group, *S. mutans* in the FPN/HH_2_O_2_ group exhibited distinct structural damage, including collapsed cell surfaces and irregular morphology (Figure 2e) aligning with previous observations (Liu S. et al., 2023). In contrast, the bacterial morphology remained largely normal in both the FPN and H_2_O_2_ groups Notably, apparent adhesion between FPN and *S. mutans* was observed in both the FPN and FPN/H_2_O_2_ groups, which further induced obvious bacterial aggregation. It was presumably because the adhesive properties of PDA enabled rapid and selective adherence of *S. mutans* to the FPN surface, facilitating localized generation of reactive ^⋅^OH and culminating in the efficient eradication of pathogenic bacteria (Cheng et al., 2023). The mechanism indicates that surface modification with adhesive polymers can enhance the spatial specificity and bactericidal potency of enzymatic catalysis.

### 3.4 Antibiofilm performance of FPN against *S. mutans* biofilms

**FIGURE 3:**
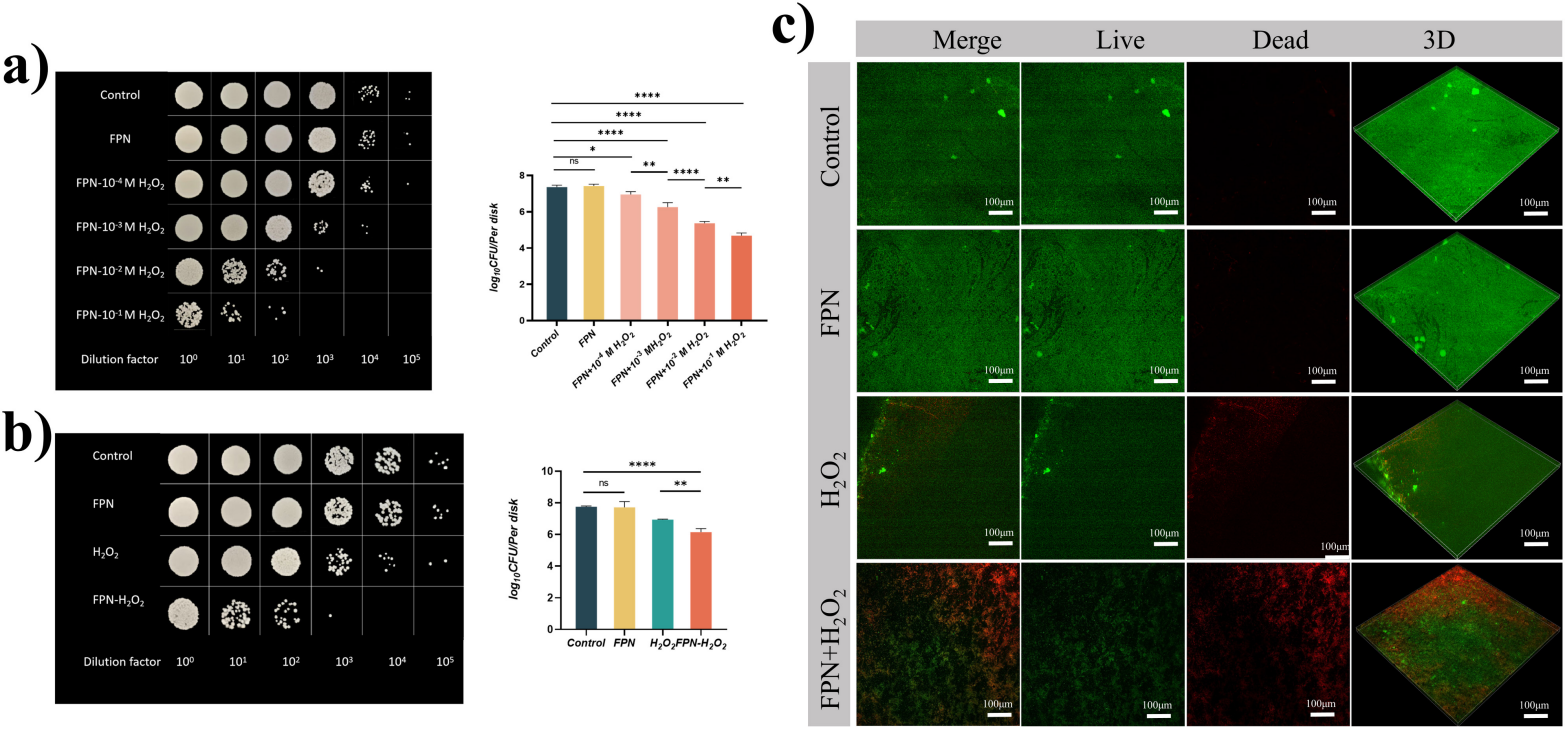
The anti-biofilm effect of FPN against *S. mutans* biofilm. **(a)** The anti-biofilm effect of different concentrations of H_2_O_2_ catalyzed by FPN on *S. mutans* biofilm. **(b)** The anti-biofilm effect of FPN combined with 10 mM H_2_O_2_ at pH = 4.5. The left images of **(a,b)** showed the CFU image and the right images showed the CFU count result. **(c)** CLSM images of bacterial biofilms after different treatments by live/dead staining. *P < 0.05, **P < 0.01, ***P < 0.001, ****P < 0.0001.

The oral cariogenic bacteria typically exist in biofilms, which affords the protection from antimicrobial drugs, such as biotics, by extracellular polysaccharides (EPS) (Wang P. et al., 2026). Given the clinical relevance of biofilm-mediated cariogenicity in oral environments, we proceeded to evaluate its biofilm-inhibitory potential by CFU and CLSM. Considering the higher resistance of biofilm than their planktonic bacteria, we first evaluated the inhibitory effects of FPN-catalyzed H2O2 at different H_2_O_2_ concentrations by CFU counts (Figure 3a). Concentrations from 10^−4^ to 10^–1^ M were examined, demonstrating that the extent of biofilm eradication improved progressively with increasing H_2_O_2_ concentrations. It was found that FPN-catalyzed 1mM H_2_O_2_ (the planktonic antibacterial concentration) at pH4.5 reduced biofilm bacteria by only 1log, without achieving a more pronounced or extensive killing effect within the biofilm, confirming that biofilms exhibit higher tolerance compared with planktonic *S. mutans*. Consequently, the H_2_O_2_ concentration was escalated to 10 mM to ensure sufficient oxidative pressure for *S. mutans* biofilm.

Further, the quantitative analysis of crystal violet staining (Supplementary Figure S2) revealed that the blank control group exhibited the highest biofilm biomass corresponding to the maximum absorbance values. While the H_2_O_2_ group demonstrated a moderate reduction in crystal violet accumulation relative to the control, the FPN/H_2_O_2_ group exhibited the most substantial decrease in both crystal formation and optical density. In addition, as illustrated in Figure 3b, FPN-catalyzed 10 mM H_2_O_2_ at pH 4.5 exerted a significant anti-biofilm effect, reducing the viable *S. mutans* count within biofilms by approximately 2 orders of magnitude. Notably, despite the elevated H_2_O_2_ concentration, it was still nearly 100-fold lower than the clinically used concentration (around 1 M) (Marshall et al., 1995; Zhang et al., 2008). These findings collectively indicate that the FPN-catalyzed system resulted in the lowest bacterial viability, demonstrating superior efficacy in the inhibition and eradication of *S. mutans* biofilms.

Further, the biofilms were examined by CLSM after live/dead staining (Figure 3c). The blank control, H_2_O_2_ alone, and FPN alone groups showed intact biofilms with abundant live bacteria (green fluorescence). In contrast, the FPN/H_2_O_2_ group exhibited substantially damaged biofilms with many dead bacteria (red fluorescence). Collectively, FPN-catalyzed H_2_O_2_ at pH4.5 demonstrated a concentration-dependent anti-biofilm effect, requiring at least 10mM H2O2 for efficacy. The anti-biofilm effect is primarily attributed to the generation of ^⋅^OH from FPN-catalyzed H_2_O_2_. In fact, harnessing ^⋅^OH to disrupt oral biofilms has been extensively documented, and H_2_O_2_, as a common source of ^⋅^OH, is widely used in oral antibacterial applications. Liu et al. employed superparamagnetic iron oxide nanoparticles to catalyze ^⋅^OH generation from H_2_O_2_, which disrupted bacterial membranes and degraded EPS (Liu et al., 2018). Hyun Koo et al. developed a bifunctional nanohybrid (Dex-IONP-GOx) that enabled GOx to oxidize glucose into H_2_O_2_, which was subsequently catalyzed by Dex-IONP to produce ROS for bacterial killing and EPS degradation (Huang et al., 2021). Shirato Midori et al. utilized UV light to catalyze 3% H_2_O_2_ to generate ^⋅^OH for enhanced anti-biofilm resistance (Shirato et al., 2023). Therefore, harnessing ^⋅^OH represents a well-established and effective strategy against bacterial biofilms.

### 3.5 The ability of FPN/H_2_O_2_ to prevent enamel demineralization *in vitro*

**FIGURE 4:**
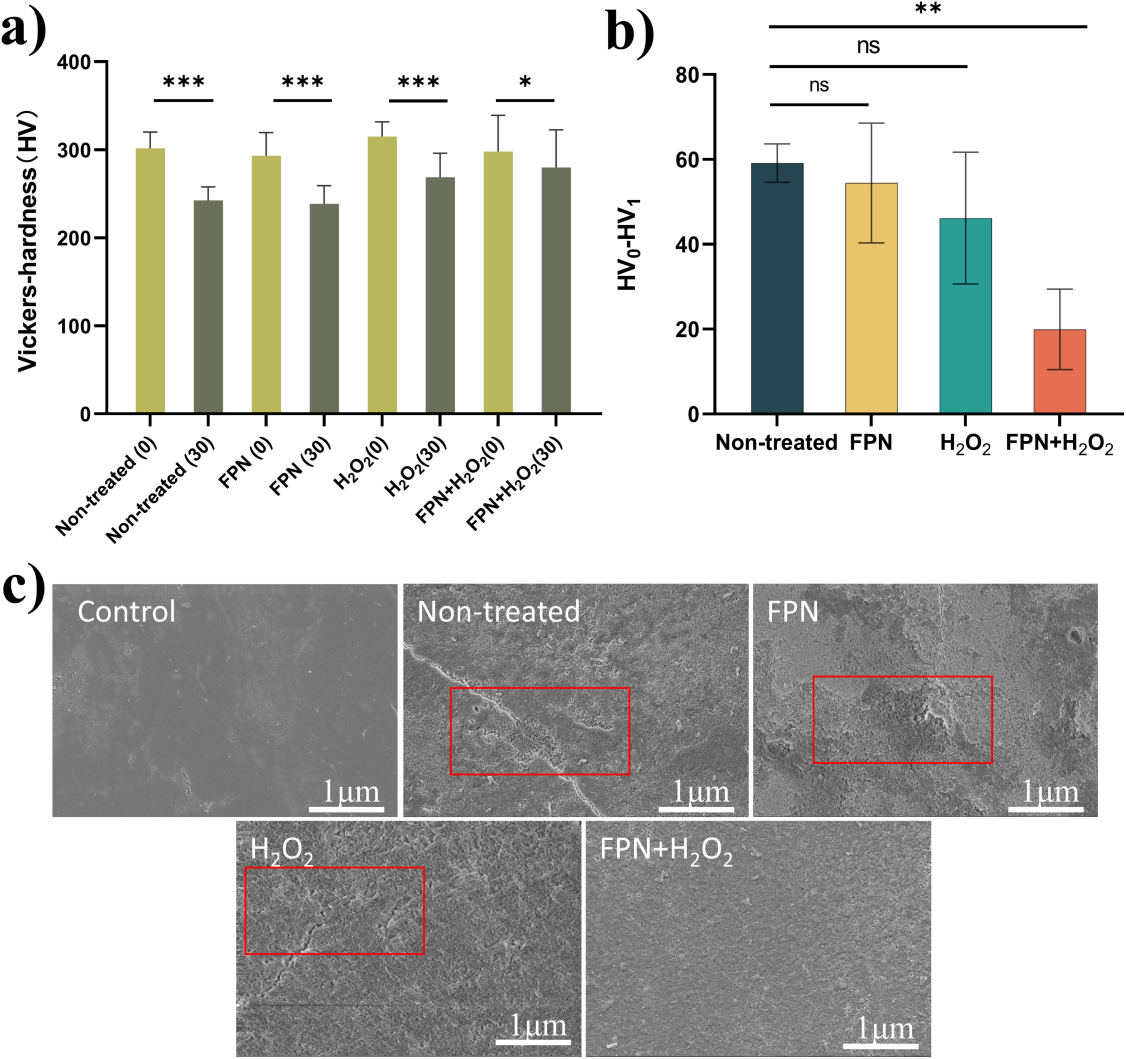
The effect of FPN combined with H_2_O_2_ for enamel surfaces. **(a)** The Vickers hardness of the enamel disks surface before incubating with *S. mutans* and 30 days later in each group. **(b)** The Vickers hardness variation of enamel disks in each group (HV_0_: the primary Vickers-hardness; HV_1_: the final Vickers-hardness). **(c)** SEM of the enamel disk surface of each group incubating with *S. mutans* for 30 days. The red boxes refer to the tooth surfaces with significant demineralization. Control group: The enamel surface without bacterial biofilms. non-treated group: The demineralization of the tooth enamel surface over a period of 30 days due to the action of biofilms. FPN group: After the formation of biofilms, enamel treated with FPN for 30 days. H_2_O_2_ group: After the formation of biofilms, enamel treated with H_2_O_2_ for 30 days. FPN + H_2_O_2_ group: After the formation of biofilms, enamel treated with H_2_O_2_/FPN for 30 days. *P < 0.05, **P < 0.01, ***P< 0.001.

Enamel demineralization in dental caries is predominantly attributed to acid production by *S. mutans*, which creates numerous microscopic fissures on the intact enamel surface, thereby facilitating further acid penetration and the proliferation of cariogenic bacteria (Gao et al., 2022). Without timely intervention, the teeth progressively develop substantial defects and cavitation, compromising tooth integrity and aesthetics. To evaluate the protective effect of the bactericidal activity of the FPN/H_2_O_2_ system on enamel, enamel disks were incubated with *S. mutans* for 30 days and treated daily with FPN/H_2_O_2_. Subsequently, the structural changes of the enamel disks were observed under SEM, and surface hardness was measured to determine whether FPN/H_2_O_2_ could mitigate demineralization and inhibit caries progression by disrupting bacterial acidogenesis and preserving enamel integrity. The Vickers hardness (HV) of enamel disks in each group was measured before (HV_0_) and after 30 days (HV_1_) of co-culture with the respective treatments (Figure 4a). The results showed that the FPN/H_2_O_2_ group exhibited no significant change in Vickers hardness over the 30-day period. In contrast, the FPN alone, H_2_O_2_ alone, and untreated control groups all showed a statistically significant decline in Vickers hardness after 30 days. Subsequently, the reduction in Vickers hardness (ΔHV = HV_0_ – HV_1_) was then compared among the groups (Figure 4b). Statistical analysis revealed that the FPN/H_2_O_2_ group had the smallest hardness reduction (∼20N mm^–2^), which was significantly different from that of the other groups (∼60N mm^–2^). Notably, variations in microhardness values directly reflect alterations in enamel mineral deposition and dissolution dynamics, thereby indicating treatment efficacy in preserving enamel structure (Mahoney et al., 2003).

Furthermore, SEM was employed to capture high-resolution surface images, a widely used technique to analyze enamel microarchitecture. In accordance with the above microhardness results, SEM observation was further performed to intuitively reveal the micromorphological changes of enamel surfaces in each group. Notably, the enamel surface of the FPN/H_2_O_2_ group displayed a smooth and intact morphology under SEM, with no observable signs of demineralization. In stark contrast, the enamel surfaces of the FPN-only, H_2_O_2_-only, and untreated control groups exhibited pronounced demineralization, characterized by dissolution of the enamel structure and widespread microfissures (Figure 4c). These SEM observations align with prior microscopic studies reporting similar enamel degradation patterns (Ding et al., 2024). Collectively, these results indicate that the FPN-H_2_O_2_ system effectively mitigates enamel demineralization, likely through synergistic bactericidal activity against S. mutans. This protective effect demonstrates significant translational potential for preventing early-stage dental caries. Therefore, these findings collectively establish FPN-catalyzed H_2_O_2_ as a compelling therapeutic modality for inhibiting enamel demineralization and advancing oral health in biomedicine.

### 3.6 Biocompatibility of FPN

**FIGURE 5:**
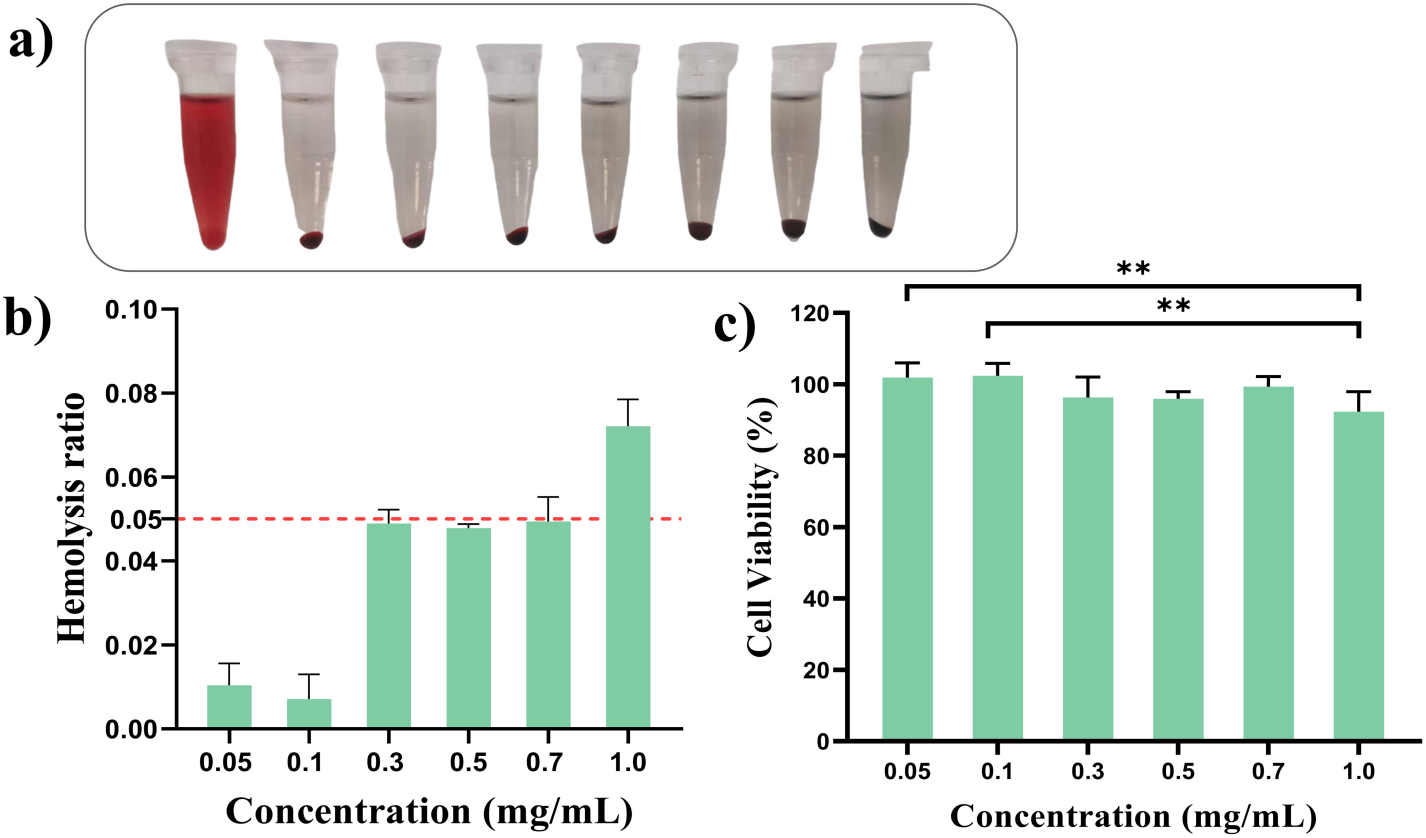
Biocompatibility of FPN. **(a)** The hemolytic images of different concentrations of FPN. **(b)** The hemolysis rate of FPN. **(c)** The cell viability rate of HGFs. **P < 0.01.

To evaluate the biocompatibility of FPN, hemolysis assay and cytotoxicity test were performed. As illustrated in Figure 5a, the hemolysis test revealed that, except for the positive control group (where the solvent is water), the red blood cells of all other groups sank to the bottom of the centrifuge tube without any discernible hemolysis. The quantitative analysis by UV-Vis spectrophotometry revealed that at relatively low FPN concentrations, the hemolysis rate was below 5%, indicating low *in vitro* toxicity (Figure 5b). Furthermore, at concentrations of 0.7 mg/mL and 1.0 mg/mL, the hemolysis rate reached 5% and above, indicating a certain degree of toxicity *in vitro*. In contrast, the CCK-8 assay showed that FPN exhibited good biocompatibility even at 0.7 and 1.0mg/mL (Figure 5c). This apparent discrepancy may be explained by the fact that FPN itself has some absorbance in the hemolysis assay, leading to an overestimation of the hemolysis rate at higher concentrations. Consequently, an initial conclusion may be drawn that FPN displayed favorable biocompatibility based on the combined results of the hemolysis test and cytotoxicity analysis. Taken together, FPN combined with H_2_O_2_ had considerable potential for application in oral biomedicine as a preventative measure against enamel demineralization.

## 4 Conclusion

In summary, ferro-polydopamine nanozymes (FPN) with remarkable POD-like activity were facilely fabricated through the simple polymerization and self-assembly process. As observed *via* SEM, FPN exhibited a flower-like structure composed of densely packed nanorods. The kinetic analysis demonstrated that FPN exhibited considerable POD-like activity, facilitating the generation of ^⋅^OH from H_2_O_2_ through catalytic processes. Notably, the antibacterial experiment demonstrated that FPN exhibited effective antimicrobial activity against *S. mutans* suspension and its bacterial biofilm by catalyzing a low concentration of H_2_O_2_ in a slightly acidic environment. Specifically, FPN demonstrated a notable anti-bacterial impact when catalyzing 1 mM H_2_O_2_ in a weakly acidic environment (pH = 4.5), which closely resembles the cariogenic environment. Furthermore, when catalyzing 10 mM H_2_O_2_, FPN exhibited a pronounced ability to eradicate *S. mutans* within the bacterial biofilm, resulting in a 2-log reduction in the bacterial population. Moreover, the potential of FPN in conjunction with H_2_O_2_ to inhibit enamel demineralization was investigated through an enamel disk experiment conducted *in vitro*. The findings indicated that FPN combined with H_2_O_2_ exhibited a favorable impact on alleviating enamel demineralization and the potential to prevent early caries. Importantly, the hemolysis test and cytotoxicity analysis indicated that FPN exhibited minimal toxicity to erythrocytes and human gingival fibroblasts. Collectively, these findings establish FPN nanozymes as a multifunctional platform that synergizes potent antibacterial activity, enamel protection, and biocompatibility, thereby offering a promising translational candidate for mitigating early dental caries while reducing reliance on high H_2_O_2_ concentrations in clinical scenarios. This integrated approach holds transformative potential in advancing oral health interventions by providing a precision medicine strategy that combines efficacy, safety, and environmental compatibility.

## Data Availability

The original contributions presented in the study are included in the article/Supplementary material, further inquiries can be directed to the corresponding author.
