# Supplementary material for: Flower-like ferro-polydopamine nanozymes with peroxidase-like activity for early caries prevention
Source: Front Microbiol. 2026 Jun 11;17:1870312. doi: 10.3389/fmicb.2026.1870312 (PMC13294468; doi:10.3389/fmicb.2026.1870312)
Supplement: Supplementary file 1 [file Table_1.docx]

Flower-Like Ferro-polydopamine nanozymes with Peroxidase-Like Activity for Early Caries Prevention

**Pei Wang^1, 2, 3#^, Ziqiang Chen^1, 2, 3#^, Yifan Liu^1, 2, 3^, Yuqing Mu^4^, Jun Guo^1, 2, 3*^**

^1^ School of Stomatology, Jiangxi Medical College, Nanchang University, Nanchang 330006, People’s Republic of China

^2^ Jiangxi Provincial Key Laboratory of Oral Diseases, Nanchang 330006, People’s Republic of China

^3^ Jiangxi Provincial Clinical Research Center for Oral Diseases, Nanchang 330006, People’s Republic of China

^4^ School of Medicine and Dentistry, Griffith University (GU), Gold Coast, Queensland 4222, Australia


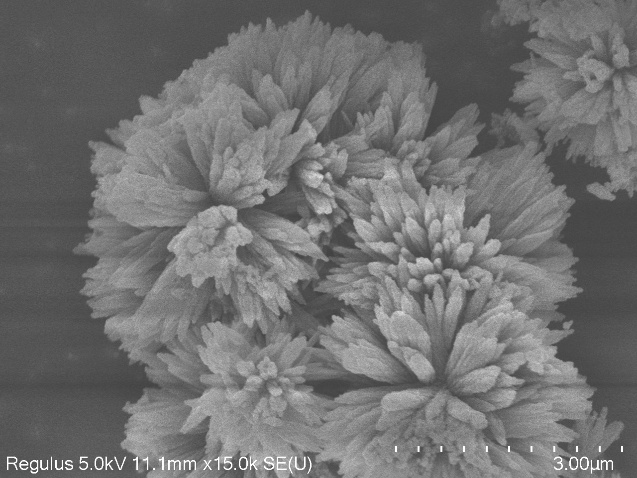


**Figure S1.** SEM image of FPN.

**Figure S2.** The crystal violet staining of *S. mutans* biofilms after various treatments.
